# Comparative Anatomy Supports the Evolution of Nocturnality in the Extinct Hawaiian Ibis *Apteribis*

**DOI:** 10.1093/icb/icaf159

**Published:** 2025-12-19

**Authors:** Sara Citron, Aubrey Keirnan, Vera Weisbecker, Helen James, Andrew N Iwaniuk

**Affiliations:** Department of Neuroscience, University of Lethbridge, Lethbridge, AB, T1J 5H2, Canada; College of Science and Engineering, Flinders University, Adelaide, SA, 5042, Australia; College of Science and Engineering, Flinders University, Adelaide, SA, 5042, Australia; Department of Vertebrate Zoology, National Museum of Natural History, Smithsonian Institution, Washington, District of Columbia, 20560, USA; Department of Neuroscience, University of Lethbridge, Lethbridge, AB, T1J 5H2, Canada

## Abstract

Evolution on islands often generates specialized lifestyles that are rarely seen in continental species. The biota on oceanic islands are, however, prone to extinctions following human colonization, resulting in an incomplete understanding of the lifestyles of species that evolved prior to colonization. For example, the Hawaiian Islands hosted a unique and diverse assemblage of endemic taxa, most of which became extinct following human colonization. Among these is *Apteribis* (Threskiornitidae), an extinct genus of flightless ibises for which nothing is known of their behaviour and ecology. To gain insight into the foraging behaviour and activity pattern of this unusual genus, we quantified their olfactory, visual, and somatosensory systems from direct measurements of skulls, CT scans, and endocasts. We then compared *Apteribis* with extant ibises with phylogeny-informed statistics to determine if they differed significantly in any of our measured traits. Our analyses show that the olfactory and somatosensory systems of *Apteribis* are comparable in size and anatomy to those of extant ibises and it was likely flexible in terms of preferred foraging habitat. In contrast, the visual system of *Apteribis* is greatly reduced in size, suggesting a nocturnal lifestyle, which is an unprecedent trait among ibises. Our data therefore suggests that *Apteribis* occupied a niche similar to that of New Zealand kiwi (*Apteryx*): nocturnal, flightless species that rely on tactile cues from its beak to detect prey. This study provides the first quantitative evidence for the evolution of a kiwi-like niche for a bird outside New Zealand, and emphasizes the remarkable diversity of avian lifestyles lost due to anthropogenic impact.

## Introduction

Studies of island biota have been fundamental to understanding many evolutionary principles, such as adaptive radiations, niche separation, and evolutionary processes on different timescales (Blondel 2000; Emerson 2002; Steadman 2006; Whittaker and Fernández-Palacios 2007; Losos and Ricklefs 2009; Warren et al. 2015). Island-dwelling birds, in particular, have played an important role in the study these principles thanks to their high ecological diversity, speciation rate, osteological record, and practical advantages for studies in the field (Grant 2001; Steadman 2006; Clegg 2010; Jezierski et al. 2024). Island-dwelling birds evolved numerous changes in relation to their mainland counterparts including plumage (Doutrelant et al. 2016; Grant 1965b), size and/or shape of body parts (Longrich and Olson 2011; Wright et al. 2016; Sayol et al. 2018; Grant 1965a), body size (Olson et al. 2009; Clegg 2010), and life history traits. One of the more notable differences between mainland and island-dwelling birds is a higher percentage of flightless species on islands (Diamond 1981; McNab 1994; Boyer and Jetz 2010; Jezierski et al. 2024). Many of these traits, especially flightlessness and lack of predator recognition, have contributed to the extinction of unique, island-dwelling species following human colonization of oceanic islands (Olson 1989; Steadman 2006; Sayol et al. 2020; Fromm and Meiri 2021).

Avian extinctions have been studied across many oceanic islands (Steadman 2006; Hume 2017; Fromm and Meiri 2021), but the Hawaiian islands are considered among the most isolated archipelagos on Earth and home to many recently extinct species. Prior to human arrival, Hawaii hosted a unique and diverse assemblage of endemic taxa including large goose-like waterfowl, a “mole duck,” and flightless ibises and geese (Olson and James 1991; Iwaniuk et al. 2009; Witmer et al. 2017; Lewis 2018), all of which went extinct following two periods of human colonization. The first was initiated after Polynesian people settled there around the 10–12^th^ century (James et al. 1987; Kirch 1911; Olson and James 1982; 1984; Steadman 1995), and the second began with European colonization beginning in the late 18^th^ century (Boyer 2008; Sayol, Steinbauer et al. 2020). Among the victims during the first period was *Apteribis*, a genus of flightless ibises that were found on Maui, Moloka’i, and Lānaʻi (Olson and James 1991; Olson and Wetmore 1976). Despite being first described about 50 years ago and abundantly represented in the Holocene fossil record with hundreds of specimens being referred to this genus, little is known about the behaviour or ecology of the two described species (*Apteribis glenos* and *A. brevis*). The two recognized species resemble each other in postcranial and cranial osteology, but differ in body size and bill length (Olson and James 1991). Based on post-cranial elements, both *Apteribis* were flightless and otherwise appear to be superficially similar to extant ibises (Olson and James 1991). Apart from the species descriptions, the extent to which *Apteribis* was similar to (or differed) from other ibis species has remained untested and their ecological niche uncertain.

Key to understanding the ecological niche of *Apteribis* species is determining their activity pattern (i.e., what time of day they were active) and foraging behaviour, both of which are a correlated with the sensory systems. The anatomy of the sensory organs and brain of an animal are typically correlated with the acuity and/or sensitivity of its senses (e.g., Catania 2005; Caves et al. 2018; Hall et al. 2009; Iwaniuk and Wylie 2020; Wackermannová et al. 2016). For example, bird species that rely heavily on tactile cues for foraging have more tactile receptors on the bill (Berkhoudt 1979; Cunningham et al. 2010a; du Toit et al. 2024; du Toit et al. 2022; Nebel and Elner 2005) and enlarged brain regions that process the bill’s somatosensory input (Cunningham et al. 2013; Gutiérrez-Ibáñez et al. 2010; Ziolkowski et al. 2022). In contrast, smaller eyes and optic lobes indicate a diminished reliance on vision, and are found in nocturnal birds (Martin et al. 2007). However, nocturnal or crepuscular birds can also maintain a relatively functional vision through bigger eyes, which enhance visual acuity through longer focal lengths and improve sensitivity via wider pupils and longer focal lengths (Potier et al., 2020). A behavioural trait (nocturnality) can thus be reflected in a change in anatomy (eye size). Both the relative size of the sensory organs and of the brain regions processing sensory input can provide valuable insights on the behaviour of extinct species. For instance, reduced optic lobes have been interpreted as evidence of nocturnality in the extinct elephant birds *Aepyornis maximus* and *A. hildebrandti*, and the Hawaiian mole-duck *Talpanas lippa* (Early et al. 2020a; Early et al. 2020b; Iwaniuk et al. 2009; Torres and Clarke 2018; Witmer et al. 2017). Here, we use the relationship between between skull anatomy and sensory abilities to gain new insights into the behaviour and ecology of *Apteribis*.

All extant ibises (Threskiornitidae), are tactile foragers, searching for prey by relying on mechanical and vibrational cues generated through probing into the substrate with the bill (Cunningham et al. 2010a; Cunningham et al. 2010b). Given that *Apteribis* probably also relied on tactile cues, and considering its loss of flight, this genus resembles other island-dwelling species such as kiwi (*Apteryx*) and the mole-duck (*Talpanas*), all of which are nocturnal (Martin et al. 2007; Witmer et al. 2017). This opens the possibility that *Apteribis* may also have been nocturnal, but we cannot predict if they have enlarged olfactory regions like kiwi or undergone expansion of somatosensory input to the bill. Using high-resolution X-ray computed tomography (CT) and measurements of skeletal specimens, we quantitatively compared *Apteribis* with extant ibises focusing on the anatomy of the sensory organs, nerves, and brain regions involved in visual, somatosensory, and olfactory processing.

## Materials and methods

### Specimens

We CT-scanned 29 skulls representing 18 ibis species, including one *Apteribis sp*. skull from Maui (USNMPAL377837). Specimen numbers, scanner type, resolution, and other details are available in the supplementary material (Table S1). To broaden our sampling of individuals and species for at least some of our measurements, we also made anatomical measurements directly from 165 skulls of 25 ibis species (Table S2). Included in these 165 skulls are 40 *Apteribis* specimens: one *A. brevis* from Maui; one *A. glenos* from Molokai; and 38 *A. sp* from Maui (from the caves of Auwahi, Puu Naio, Kahawaiha Papa, Huki-huki, Puu Makua, Lua Lepo, Polipoli) (Olson and James 1991) and Lanai (from Feather Cave) (Dove and Olson 2011). Details of all these specimens are also provided in the supplementary material (Table S2).

### Taxonomy

The genus *Apteribis* was introduced in 1976 by Olson and Wetmore (1976), with *A. glenos* as the sole species in the genus. The holotype of *A. glenos* is from Molokai, and a paratype was designated from Maui. Subsequently, a large number of *Apteribis* fossils were recovered from lava caves on Maui. Olson and James (1991) found a wide range of body sizes in these Maui specimens, with smaller individuals tending to occur at higher elevations. They described a second species, *A. brevis*, designating a small-bodied individual from a high elevation site as the holotype. Two species were therefore likely present on Maui: the smaller, *A. brevis*, which preferred higher elevations and moister climates, and a larger species in the drier lowlands, which was either an undescribed species or, less likely, conspecific with *A. glenos*. The two Maui species appear to overlap in range at mid-elevations. Many of the Maui specimens could not be designated to either *A. brevis* or to the undetermined lowland species, and have since been referred to only as *Apteribis sp*. from Maui.

### Anatomical measurements

To estimate the sensory abilities of *Apteribis* relative to extant ibises, we measured proxies of the sensory organs and cranial nerves from both the CT scans and skeletal specimens. Data for all specimens are provided in Tables S1 and S2.

**Fig. 1:**
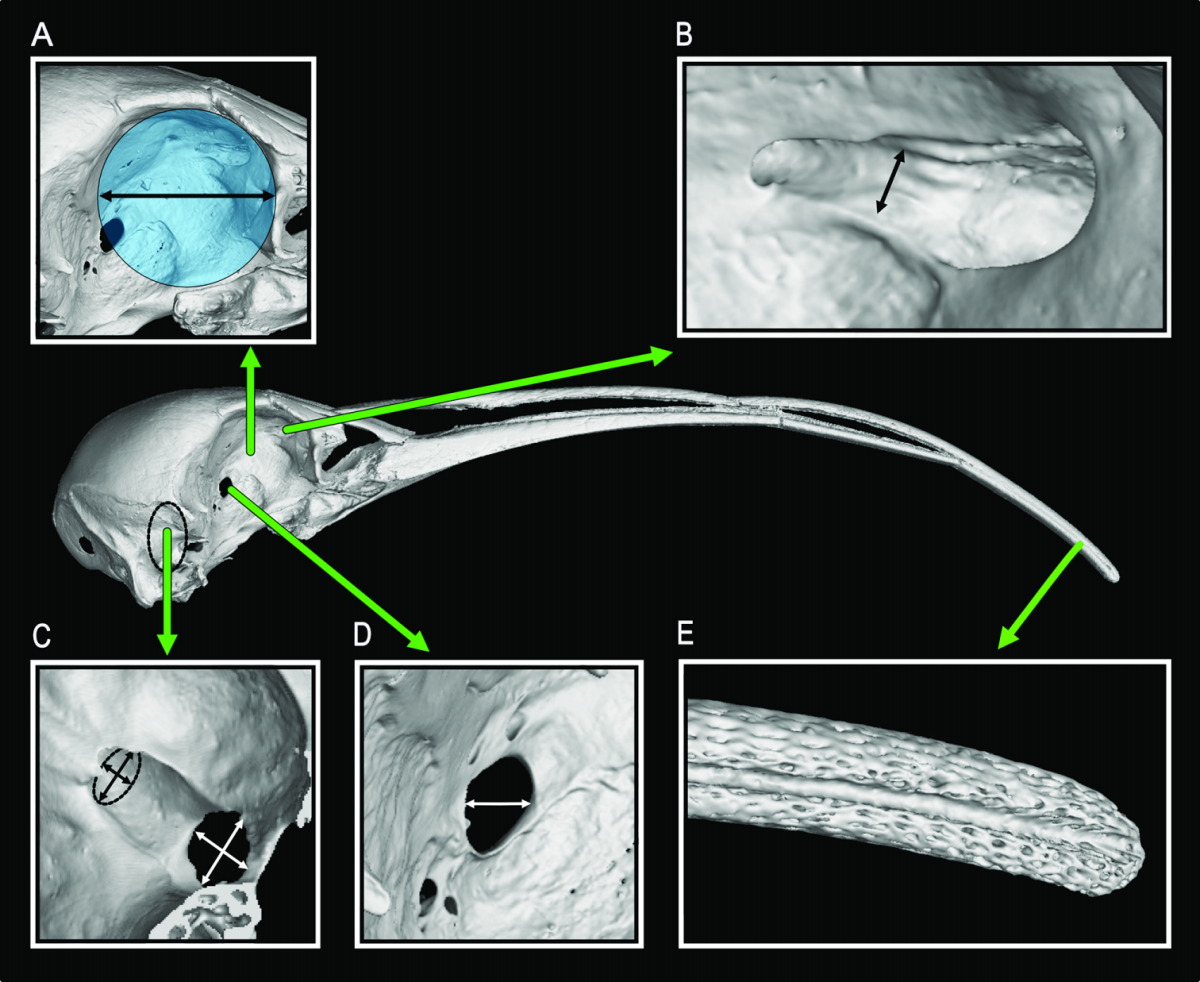
Digital reconstruction of *Apteribis sp*. skull (USNMPAL377837) illustrating the osteological measurements taken. (A) Largest circle that fits within the bony orbit. The black line with arrows indicates the circle diameter. (B) Close up of the olfactory sulcus, used as a proxy of the olfactory nerve. The black line with arrows indicates its diameter rostral to the foramina nervus olfactorii. (C) Close up of the foramen nervus ophthalmicus (black arrows) and the foramen nervus maxillomandibularis (white arrows). The area of the foramina was used to quantify the trigeminal nerves V1 (nervus ophtalmicus) and V2 + V3 (nervus maxillomandibularis), respectively. The black and white segments indicate the two perpendicular major diameters used to obtain the elliptical area of the foramina. (D) Close up of the optic foramen, with white segment showing its rostro-caudal maximum diameter. (E) Close up of the distal part of the upper bill, showing the pits of the bill tip organ.

To estimate eye size, we measured the orbit diameter of CT scanned skulls in VGstudioMax (v. 2024.2.1, Hexagon Manufacturing Intelligence Inc., Huntersville, NC, USA) by measuring the diameter of the largest circle that would fit within the bony orbit (Fig. 1A). To verify that the eye completely fills the bony orbit, we dissected two ibis specimens (*Eudocimus ruber* USNM505779 and *Threskiornis aethiopicus* USNM542171). As shown in supplementary Fig. S1, the eyes of both specimens are close to the edges of the bony orbits, supporting the use of orbit diameter as an estimate of eye size (see also Hall and Ross 2007).

The optic nerve projects from the eye to the brain and passes through the optic foramen. The optic foramen can therefore be used as an indicator of the amount of visual information (i.e., number of retinal ganglion cell axons) projecting to the optic tectum and other visual regions of the brain (Hall et al. 2009). In ibises the optic foramen is fused with the interorbital foramen (Fig. 1) and it is often incompletely ossified in the dorso-ventral axis (Hall et al. 2009). We therefore measured only the maximum rostro-caudal diameter of the optic foramen directly on the skulls with dial calipers to the nearest 0.01 mm (Fig. 1D) as measure of optic foramen size.

The bill tip organ is a specialized sensory area of the distal tip of the bill tip (Gentle and Breward 1986). In beak probing birds (e.g., kiwis, ibises, shorebirds) and waterfowl, the bill tip organ enables the detection of prey buried in the substrate, or otherwise not visible (Cunningham et al. 2007; Cunningham et al. 2010a; Cunningham et al. 2010b; Cunningham et al. 2009; Cunningham et al. 2013; du Toit et al. 2024; du Toit et al. 2022; Gottschaldt and Lausmann 1974; West et al. 2022). The bill tip organ is an aggregation of bony pits that house mechanoreceptors sensitive to vibrations and pressure changes (du Toit et al. 2022). Thus, species with more bill pits likely have greater somatosensory acuity and sensitivity than other species because they have more receptors (Cunningham et al. 2013). In ibises specifically, the number of pits reflects the primary foraging habitat; species that forage primarily on drier substrates (e.g., grassland) have fewer pits whereas species foraging primarily in aquatic habitats (e.g., marshes, swamps, riverine areas) have the highest number of pits (Cunningham et al. 2010a; du Toit et al. 2022). Between these extremes, are species that forage primarily on intermediate substrates (e.g., moist soils, water < 3 cm deep) or across a range of habitat types. To estimate somatosensory abilities, and by extension some aspects of foraging behaviour in *Apteribis*, we therefore counted the number of pits on the bill. We did this directly on skulls and only on specimens where the rhamphotheca had been removed (i.e., looking like Fig. 1E). We used residual-free tape on the specimens to subdivide the surfaces in sections, and then manually counted the pits under a dissecting microscope (10x magnification) with a tally counter. Pits were counted on both the outer and inner surface of the bill. The extent of the pit-covered area differs slightly in the different bones; it typically ends more rostrally on the premaxillae, more caudally on the maxillae and even more caudally on the mandibles. Therefore, we expressed the length of the bill tip organ as the distance from the distal tip of the bill to the caudal-most pits, averaged among premaxillae, maxillae, and mandibles. Pit density was then calculated by dividing the total number of pits by the length of the bill tip organ.

The trigeminal nerve carries information from the bill tip organ, as well as the face, to the brain via three branches: V1 (nervus ophthalmicus), V2 (nervus maxillaris) and V3 (nervus mandibularis) (Berkhoudt 1979; Crole and Soley 2016; Dubbeldam and Veenman 1977; Gentle 1989; Pettigrew and Frost 1985). V1 enters the braincase via the foramen nervus ophthalmicus, while V2 and V3 merge before entering via the foramen nervus maxillomandibularis (Fig. 1C) (Baumel 1993). To estimate the amount of tactile input from the face (V1) and bill tip (V2/3), we measured the areas of these two foraminae from CT scans in VGStudioMax, as they can be best seen from inside the endocranial cavity.

Last, as an estimate of olfactory input, we measured the width of the olfactory sulcus (Fig. 1B). The olfactory sulcus is a distinct groove through which the olfactory nerve passes from the olfactory epithelium in the beak to the olfactory bulbs in the brain. It is located on the interorbital septum, at the junction between the mesethmoid and frontal bones (Baumel 1993). We quantified the olfactory sulcus by measuring its minimum dorso-ventral diameter rostrally to the foramina nervus olfactorii using dial calipers to the nearest 0.01 mm (Fig. 1B).

### Endocast reconstructions

We reconstructed endocasts by first importing CT scans of skulls in VGstudioMax and converting them into meshes. We then generated endocast meshes using *endomaker* (Arothron package, (Profico et al. 2020) in R (2024, v. 4.4.2, (Team 2010). The endocast mesh was then refined with Blender software v. 4.3 (Blender Foundation, Amsterdam, The Netherlands) by closing holes in the mesh, and removing artifacts, blood vessels and neurovascular bundles, following (Balanoff et al. 2016) and (Keirnan et al. 2025). Once completed, we extracted the total endocast volume, total endocast surface area, and measurements of two brain regions: the optic lobes and olfactory bulbs. The optic lobes house the optic tectum, the primary target of the optic nerve in birds (Butler and Hodos 2005; Mpodozis et al. 1995). The surface area of the optic lobes is correlated with the volume of the optic tectum (Early et al. 2020a) such that the optic lobes are a reasonable proxy of the amount of visual input to the brain in most birds. To measure the optic lobes surface, we created a mesh of the region delimitated by the fossa tecti mesencephali, as in (Early et al. 2020a). This was done by using the freeform surface tools and the extract object properties function in VGstudioMax.

The olfactory bulbs, the first site of olfactory processing in the brain (Butler and Hodos 2005), are not accurately segmented by *endomaker*. We therefore manually segmented the olfactory bulbs in VGStudioMax with the Geometry module and then merged the generated mesh with the rest of the endocast, performing a best-fit alignment. In ibises, the pallium often extends dorsally, partially covering the caudal part of the olfactory bulbs, so that the commonly used Cobb’s ratio (Cobb 1968) is difficult to apply accurately. To quantify the olfactory bulbs, we therefore manually isolated them and measured their total volume. We defined the caudal boundary of the bulbs by examining the endocasts in lateral view and placing a plane that connected the dorsocaudal-most and ventrocaudal-most points at which the olfactory bulbs remained distinguishable. We then converted this ROI mesh into a volumetric object and extracted its volume in VGStudioMax.

### Statistical analysis

For some of our measurements, we compared values relative to body mass. Body mass data for most extant species were obtained from (Dunning Jr 2007), using the average of both sexes as representative of the species. The one exception was *Cercibis oxycerca*, for which body mass data was obtained from the AVONET database (Tobias et al. 2022). The body mass of *Apteribis* was estimated for both *Apteribis brevis* and *A. sp*. in (Iwaniuk et al. 2004). However, the specimens of *A. glenos* lack any preserved measurable traits that would allow for body mass estimation, so all analyses of body mass excluded this species.

We ran all the statistical analysis in R Core Team (2024, v. 4.4.2, (Team 2010). We used phylogenetic analyses of covariance (pANCOVA) as implemented in *evomap* (Smaers and Mongle 2014) to test whether *Apteribis* differs significantly from other Threskiornitidae for all measured traits. To compare the different brain regions, we subtracted the region of interest (optic lobes or olfactory bulbs) from the scaling variable (endocast volume or surface area) (Deacon 1990). We used phylogenetic generalized least squares (PGLS) as provided in the *nlme* package (Pinheiro et al. 2012) of log-transformed data to account for phylogenetic proximity when estimating the expected covariance in the data (Garamszegi 2014). A phylogeny was obtained from birdtree.org (Jetz et al. 2014), but this did not include *Theristicus branickii, Threskiornis bernieri*, or *Apteribis* species. Following (West 2014), we placed *Theristicus branickii* as a sister taxon to *Theristicus melanopsis*, and following (Andrianarimisa and Razafimanjato 2008) we placed *Threskiornis bernieri* as sister taxon to *Threskiornis aethiopicus* (Supplementary Fig. 2). We placed *Apteribis* as sister taxon of *Eudocimus*, following (Fleischer and McIntosh 2001). Currently, there is no consensus regarding the placement of spoonbills (*Platalea*) and of some South American species (*Plegadis, Theristicus*) (Ramirez et al. 2013) relative to other genera and species. We therefore estimated the influence of phylogenetic uncertainty using the function “tree_phylm” in the *sensiPhy* package in R (Paterno et al. 2018). This analysis confirmed that *Apteribis*’ results are not affected by phylogenetic uncertainty within Threskiornithidae (all p < 0.004, Supplementary materials). Despite the acknowledged uncertainty in phylogeny, we evaluated the phylogenetic signal of our data using Pagel’s λ (Pagel 1999) as implemented in the *ape* package (Paradis and Schliep 2019), with the understanding that the relatively small number of species also limits the statistical power of λ (Pearse et al. 2023). Some of the analyses generated a negative λ, so we reanalyzed the data without including phylogenetic information, and that always confirmed the result reported here between *Apteribis* and other ibises. For each analysis, we pruned the tree to match the number of species for which data were available.

## Results

### Visual system

**Fig. 2:**
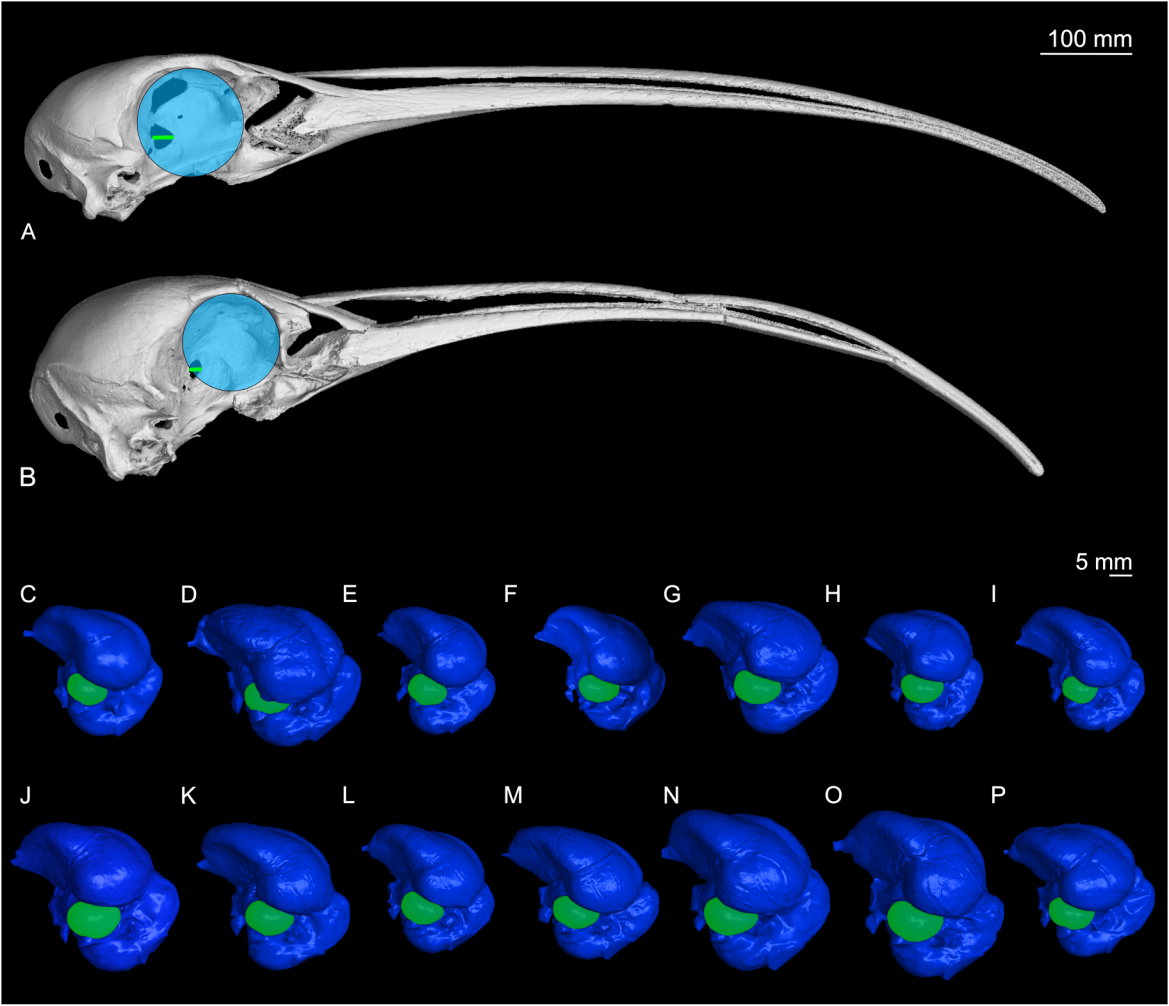
Top: digital reconstructions of the skulls of (A) American White Ibis (*Eudocimus albus* skull ROM112456), and (B) *Apteribis* sp. (USNMPAL377837), with azure circle highlighting the orbit and green segment highlighting the optic foramen. Bottom: endocasts of (C) Roseate Spoonbill (*Platalea ajaja*), (D) *Apteribis sp*., (E) Hadada Ibis (*Bostrychia hagedash*), (F) Sharp-tailed Ibis (*Cercibis oxycerca*), (G) American White Ibis (*Eudocimus albus*), (H) Northern Bald Ibis (*Geronticus eremita*), (I) Madagascar Ibis (*Lophotibis cristata*), (J) Green Ibis (*Mesembrinibis cayennensis*), (K) Crested Ibis (*Nipponia nippon*), (L) Bare-faced Ibis (*Phimosus infuscatus*), (M) White-faced Ibis (*Plegadis chihi*), (N) Red-naped Ibis (*Pseudibis papillosa*), (O) Black-faced Ibis (*Theristicus melanopis*), and (P) Straw-necked Ibis (*Threskiornis spinicollis*). The green-marked brain region highlights the optic lobe. Note the reduced optic system of *Apteribis* for all three traits: orbits, optic foramen, and optic lobes.

**Fig. 3:**
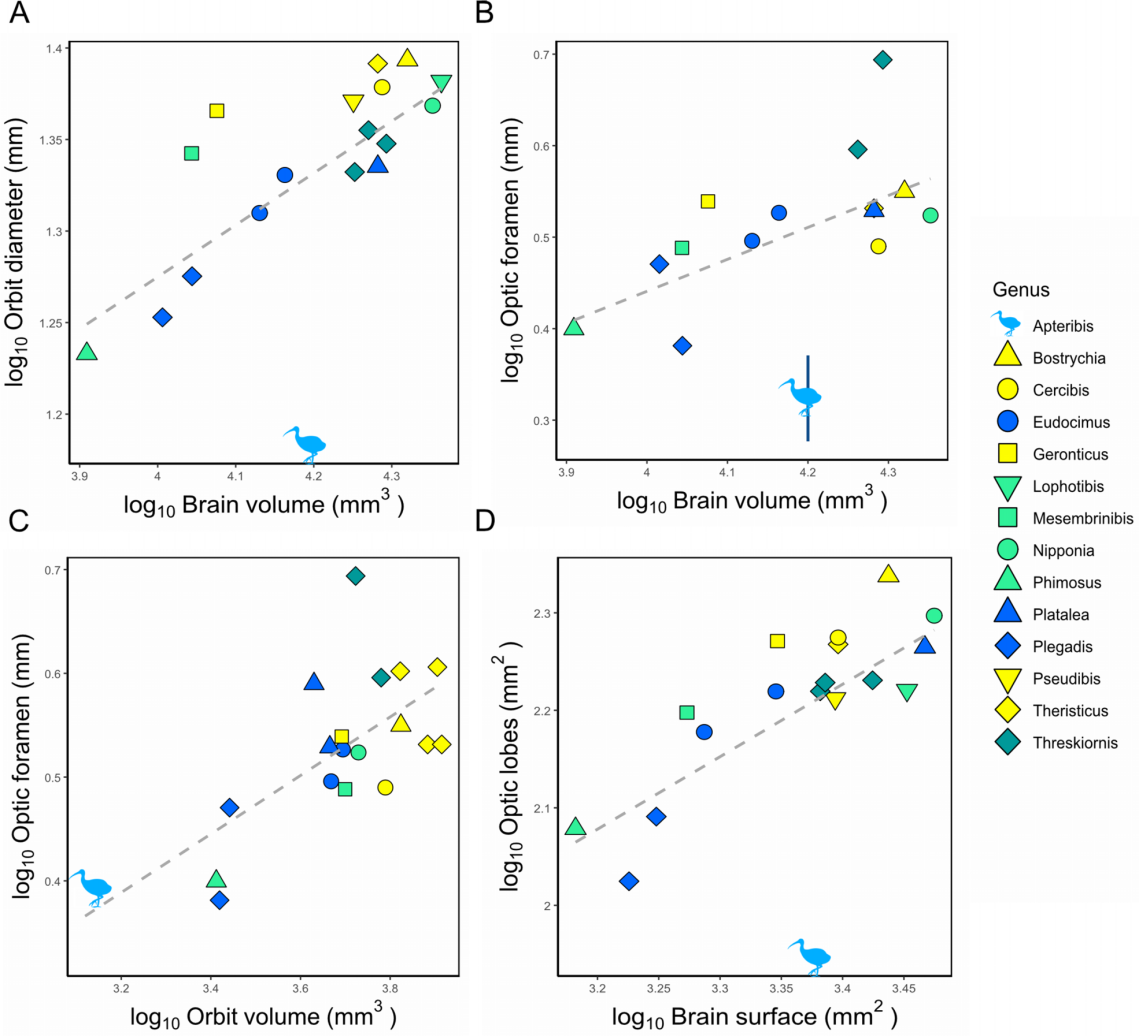
(A) Scatterplot of the log-transformed orbit diameter (mm) plotted against the log-transformed brain volume (mm^3^). (B) Scatterplot of the log-transformed optic foramen size (mm) plotted against the log-transformed brain volume (mm^3^). Vertical segment shows *Apteribis’* intrageneric standard deviation. (C) Scatterplot of the log-transformed optic foramen size (mm) plotted against the log-transformed orbit volume (mm^3^). (D) Scatterplot of the log-transformed optic lobes surface area (mm^2^) plotted against the log-transformed rest of the brain surface (mm^2^). Legend for the symbols is on the right side, with colors indicating different preferred feeding habitat, combining information from (Hancock et al. 2010) and (Billerman et al. 2022). Yellow = dry soil (soft ground of dry, arid and semi-arid areas), light green = variable (moist soil or water always less than 3 cm deep, damp areas with deep leaf litter, edges of streams and lagoons, swampy habitat, marshes, humid forest, but also dry forest), dark green = extremely variable (margins of freshwater wetlands, lagoons, intertidal areas grasslands, inundated areas with water always less than 3 cm deep, but also recently burnt areas, semi dry grasslands) blue = submerged (water deeper than 3 cm, but also shallow waters and very damp mud in coastal and interior freshwater marshes, swamps, rivers-edges). Dashed grey line represents the linear regression weighted to account for the phylogeny.

Digital reconstructions of *Apteribis* sp. (USNMPAL377837) and American White Ibis (*Eudocimus alba*, ROM112456) skulls are shown in Fig. 2, with the area of the orbit highlighted. Below the skull reconstructions are lateral views of the endocasts of all ibis genera examined in this study, with the optic lobes highlighted. Based on lateral views of the skulls and endocasts, the visual system of *Apteribis* (orbits and optic lobes) appear to be smaller than in all the other genera. Our quantitative analyses support this observation across all three measured anatomical traits of the visual system. First, the skull of *Apteribis sp*. (USNMPAL377837) has significantly smaller orbits than the other ibises relative to brain volume (F = 24.75, p = 0.0002, λ=0.43) (Fig. 3A). The difference is retained when controlling for body mass (F = 48.88, p= <0.0001, λ=-0.21) or basicranial length (F = 26.05, p = 0.0001, λ=-0.07). As shown in Fig. 3A, *Apteribis*’ orbits are 2.5 times smaller than the American White Ibis, a species of comparable brain size and putatively a close relative of *Apteribis* (Fleischer and McIntosh 2001). Similarly, the orbits of *Apteribis* are 5 times smaller than species of similar body mass (e.g., *Geronticus eremita*), and 2 times smaller than species of similar basicranial length (*Phimosus infuscatus*).

Second, the difference in visual system between *Apteribis* and other ibises also extends to the optic foramen; *Apteribis brevis* and *A. sp* have a significantly smaller orbit foramina than other ibises relative to brain volume (F = 9.97, p = 0.0083, λ=0.30, Fig. 3B). Again, in comparison with the American White Ibis and Scarlet Ibis (*Eudocimus albus* and *E. ruber*), two closely related species of similar brain size, the optic foramen of *Apteribis* is 1.4 times smaller. However, when scaled for orbit volume, the optic foramen in *Apteribis* is not proportionally smaller than in other ibises

(F = 0.00004, p = 0.99, λ=0.54, Fig. 3C).

Third, there are corresponding reductions in the surface areas of the optic lobes of *Apteribis*. Relative to the rest of the endocast surface area, *Apteribis sp*. has significantly smaller optic lobes than the other ibises (F = 38.14, p < 0.0001, λ= 0.48). Again, in comparison with *Eudocimus* species, the optic lobes of *Apteribis* are almost two times (1.8x) smaller (Fig. 3D). Thus, there is an overall reduction in the relative size of the *Apteribis* visual system that extends from the orbits to optic foramen and to the optic lobes.

### Somatosensory system

**Fig. 4:**
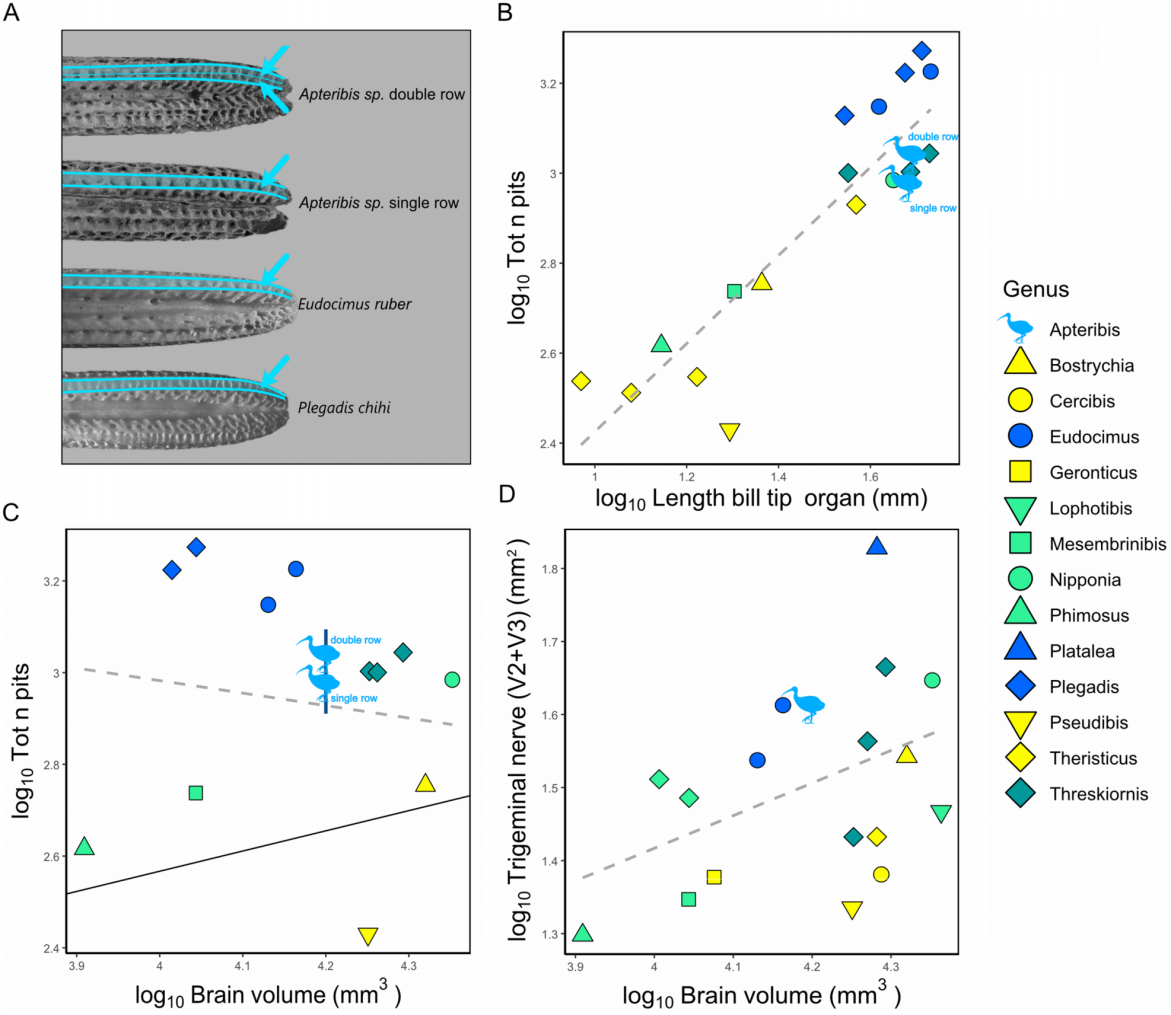
(A) Pictures of the mandibles in dorsal view showing the oral surface in *Apteribis sp., Eudocimus ruber* and *Plegadis chihi*. Note the double row of pits present in *Apteribis sp*.—double row. Pictures not in scale. (B) Scatterplot of the log-transformed total number of pits in the bill plotted against the log-transformed length of the bill tip organ (mm). (C) Scatterplot of the log-transformed total number of pits plotted against the log-transformed brain volume (mm^3^). Vertical blue segment shows *Apteribis’* intrageneric standard deviation. Black line represents the regression line not based on phylogeny. Note the difference between the two type of *Apteribis*. (D) Scatterplot of the area of the log-transformed maxillomandibularis branch (V2 + V3) of the trigeminal nerve (mm^2^) plotted against the log-transformed brain volume (mm^3^). Legend for the symbols is on the right side, with colors indicating different preferred feeding habitat, combining information from (Hancock et al. 2010) and (Billerman et al. 2022). Yellow = dry soil (soft ground of dry, arid and semi-arid areas), light green = variable (moist soil or water always less than 3 cm deep, damp areas with deep leaf litter, edges of streams and lagoons, swampy habitat, marshes, humid forest, but also dry forest), dark green = extremely variable (margins of freshwater wetlands, lagoons, intertidal areas grasslands, inundated areas with water always less than 3 cm deep, but also recently burnt areas, semi dry grasslands) blue = submerged (water deeper than 3 cm, but also shallow waters and very damp mud in coastal and interior freshwater marshes, swamps, rivers-edges). Dashed grey line represents the linear regression weighted to account for the phylogeny.

Mechanosensory receptor pits in the bill tip organ could be counted on four (4) complete *Apteribis* specimens (i.e., preserving intact maxillae and mandibles), on six (6) specimens preserving only the maxillae, and on nine (9) specimens preserving only the mandibles. The majority of the *Apteribis* specimens have bill tips qualitatively similar to that of other ibis species. However, the lingual side of the mandibles of some *Apteribis sp*. specimens had a double-row of pits, a feature absent in all other ibises (Fig. 4A). Based on the number of mandible pits, we could split the *Apteribis* specimens into three groups: *A. glenos*, which has the fewest number of pits (mean = 50, SD = 1.41, n = 2); *A. sp*. with a single row of pits (mean = 98 pits, SD = 14.95, n = 8); and *A. sp*. with a double row of pits (154 pits, n = 1). The holotype of *Apteribis brevis* (Olson and James 1991) lacks the distal part of the mandible, but judging from the caudal portion, it likely had only a single row of pits. It is unclear whether two vs. one row of pits is a species-level delimiter in Maui’s *Apteribis* because of a lack of post-cranial elements that could be associated conclusively with the mandible specimens.

It was reported previously that the number of pits per unit length of the bill tip organ (i.e., pit density) is not informative because it is similar across ibis species (du Toit et al. 2022). Our data corroborates that finding; all species we sampled have similar pits densities (Fig. 4B), but it is noteworthy that the number of pits and the length of the bill tip organ show a positive correlation, which is associated with differences in habitat use; species foraging in wetter habitats tend to have more bill pits and longer bill tip organs. *Apteribis* does not differ significantly in pit density from other ibises (F = 1.86, p = 0.19, λ=0.97) and overlaps with *Threskiornis* and *Nipponia*. Similarly, the total number of pits in *Apteribis* did not differ statistically from other ibises when controlled for brain volume (F = 0.01, p = 0.97, λ=1.18), body mass (F = 0.04, p = 0.84, λ=1.68) or basicranial length (F = 0.02, p = 0.88, λ=1.17). However, in terms of absolute pit count, *Apteribis* is at the higher end of the range, with values comparable to *Threskiornis* and *Nipponia* and lower than *Eudocimus* (Fig. 4B, C). The one spoonbill we measured (*Platalea ajaia*) was excluded from this analysis and graph because the lateral expansion of its bill significantly increases the surface area of the bill tip organ dramatically. This morphology, along with the resulting total number of sensory pits, is not directly comparable to that of the other Threskiornithidae species, which all have tapered bills.

Given the lack of difference in bill pits across species, it was not surprising that the trigeminal nerve in *Apteribis* also did not differ from other ibises. The ophthalmic branch (V1) did not differ in size relative to brain volume (F = 0.47, p = 0.50, λ=-0.24), body mass (F = 0.75, p = 0.40, λ=-0.32), or basicranial length (F = 0.64, p = 0.44, λ=-0.185764). Similarly, the maxillomandibularis branch (V2 + V3) did not differ in size relative to brain volume (F = 0.81, p = 0.38, λ=0.45, Fig. 4D), body mass (F = 1.16, p = 0.3, λ=0.42), or basicranial length (F = 0.03, p = 0.86, λ=-0.009). When all of the branches are considered together (combining the areas of V1 and V2 + V3), the trigeminal nerve is again not significantly different from the other ibises when controlled for brain volume (F = 0.80, p = 0.38, λ=-0.12), body mass (F = 1.29, p = 0.28, λ= 0.13), or basicranial length (F = 0.1, p = 0.75, λ= -0.03). The dimensions of the trigeminal foraminae of *Apteribis* remained similar to *Eudocimus* in all instances. Note that in Fig. 4D, the Roseate Spoonbill (*Platalea ajaja*) has a trigeminal nerve 1.5 to 2.8 times larger than species with similar brain size and this reflects the aforementioned expansion of the bill tip organ of their spoon-shaped bills.

### Olfactory system

**Fig. 5:**
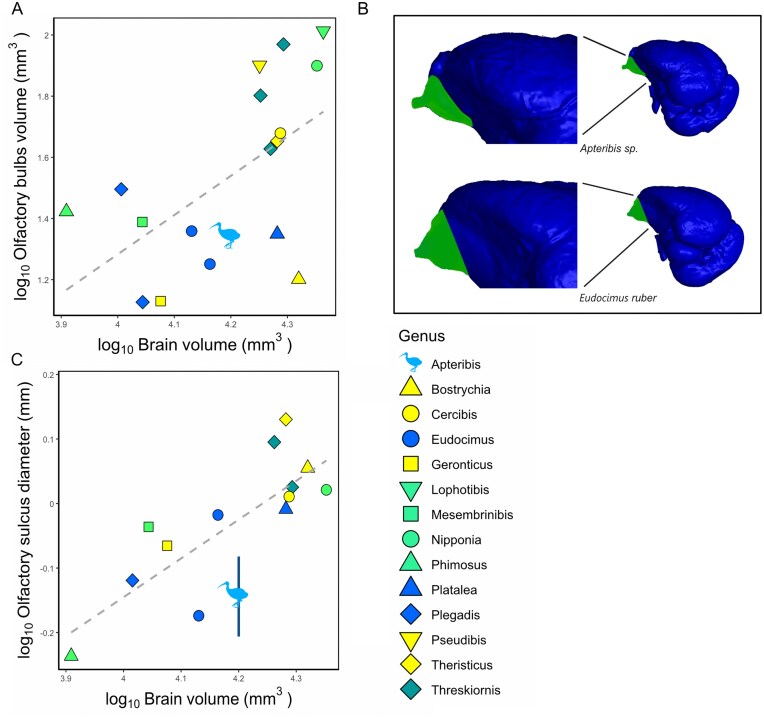
(A) Scatterplot of the log-transformed area of the olfactory bulbs (mm^3^) plotted against the log-transformed area of the rest of the brain volume (mm^3^). (B) Digital reconstruction of *Apteribis* sp. (USNMPAL377837) and *Eudocimus alba* (ROM112456) endocasts, highlighting the olfactory bulbs. (C) Scatterplot of the log-transformed diameter of the olfactory sulcus (mm) plotted against the log-transformed brain volume (mm^3^). Vertical segment shows *Apteribis’* intrageneric standard deviation. Legend for the symbols is on the right side, with colors indicating different preferred feeding habitat, combining information from (Hancock et al. 2010) and (Billerman et al. 2022). Yellow = dry soil (soft ground of dry, arid and semi-arid areas), light green = variable (moist soil or water always less than 3 cm deep, damp areas with deep leaf litter, edges of streams and lagoons, swampy habitat, marshes, humid forest, but also dry forest), dark green = extremely variable (margins of freshwater wetlands, lagoons, intertidal areas grasslands, inundated areas with water always less than 3 cm deep, but also recently burnt areas, semi dry grasslands) blue = submerged (water deeper than 3 cm, but also shallow waters and very damp mud in coastal and interior freshwater marshes, swamps, rivers-edges). Dashed grey line represents the linear regression weighted to account for the phylogeny.

*Apteribis* shows no evidence of changes in the relative size of the olfactory system compared with other ibises (Fig. 5). The olfactory sulcus in *Apteribis sp*. is not significantly different in diameter when controlled for brain volume (F = 1.56, p = 0.24, λ=-0.11), body mass (F = 3.63, p = 0.07, λ=-0.22), or basicranial length (F = 0.78, p = 0.39, λ=-0.34) (Fig. 5C). Similarly, the olfactory bulb’s volume in *Apteribis sp*. is not significantly different from other ibises when controlled for brain volume (F = 0.13, p = 0.73, λ=0.79) (Fig. 5A). For both the olfactory sulcus and bulbs, *Apteribis* was most similar in relative dimensions to *Eudocimus* (Fig. 5).

## Discussion

Based on our analyses, *Apteribis* has evolved a somewhat unique sensory ecology compared with other ibises, primarily characterized by a reduced reliance on sight. The striking reduction of the visual system in *Apteribis* encompasses orbits, optic foramen, and optic lobes. The other sensory modalities examined (olfaction and somatosensation from the bill) do not, however, differ between *Apteribis* and other ibises. *Apteribis* can therefore be considered similar to its likely relatives, the *Eudocimus* ibises, but with a reduced reliance on vision. These findings have implications for understanding the ecological niche that *Apteribis* occupied as well as for broader evolutionary patterns among island dwelling birds.

As discussed in our results, key parameters of the visual system of *Apteribis* are reduced between 1.4–2.5x times relative to species of comparable brain size (Figs. 3A, B, D). Orbit size is closely associated with eye size in ibises (Supplementary Fig. 1), and smaller eyes result in lower visual acuity (Caves et al. 2024; Martin 1993). In absolute terms, *Apteribis*’s eyes are smaller than other ibises: its orbit length (proxy for eye size) is 15.25 mm, compared with a mean of 21.96 mm in other members of the Threskiornithidae. An orbit length of ca. 15 mm occurs in both photopic and scotopic birds species and therefore does not on its own indicate nocturnality (Hall 2008). Across most avian groups, scotopic species have absolutely larger eyes than in photopic species; however, this pattern is mostly evident when scaled for head or body size. Consequently, relative eye size is more informative than absolute measurements when assessing activity pattern. Similarly, relatively narrower optic foramina imply thinner optic nerves, also reflecting lower visual resolution because the nerve is comprised of fewer retinal ganglion cell axons (Hall et al. 2009). Finally, optic lobes contain the optic tectum, the primary target of the optic nerve in most birds (Butler and Hodos 2005; Mpodozis et al. 1995), and smaller optic lobes are associated with lower visual acuity as well (Fröhlich et al. 2024; Wylie et al. 2009). Collectively, these combined reductions strongly suggest a decrease in visual acuity in *Apteribis*. While this is not surprising for a probing bird, the greater reduction compared to other ibises implies a significant change in lifestyle relative to extant species. Among extant birds, similar reductions in the visual system have been documented in kiwi (*Apteryx* sp.) (Martin et al. 2007), kakapo (*Strigops habroptilus*) (Corfield et al. 2011), and night parrot (*Pezoporus occidentalis*) (Iwaniuk et al. 2020), all of which are nocturnal. The degree of reduction varies among these species; it is limited to post-retinal structures in the two parrots, but extends to the entire visual pathway in kiwi (Corfield et al. 2011; Iwaniuk et al. 2020; Martin et al. 2007). The visual system reduction in *Apteribis* is between that of the parrots and kiwi, thereby supporting a nocturnal activity pattern. A similar conclusion was reached for the Hawaiian mole-duck (*Talpanas lippa*) based on skull morphology and optic foramen size (Witmer et al. 2017). Other extinct taxa with more moderate reductions in parts of the visual system have also been interpreted as active in crepuscular or scotopic niches (i.e., the elephant birds, *Sylviornis neocaledoniae*, and potentially some Moa species) (Early et al. 2020b; Johnston and Mitchell 2021; Riamon et al. 2022; Torres and Clarke 2018). Notably, if *Apteribis* was mainly active at night, this would be an unprecedented trait among ibises. Although nocturnal or crepuscular foraging has been reported in the spoonbills (*Platalea*) (Fasola and Canova 1993; McGinness et al. 2025) and the Crested Ibis (*Nipponia nippon*) (Li et al. 2025), these behaviors appear to be flexible and opportunistic. The ibis clade lacks strictly nocturnal or predominantly crepuscular species (Rojas et al. 1997; Billerman et al. 2022; Hancock et al. 2010).

*Apteribis*’ closest relative is *Eudocimus* (Fleischer and McIntosh 2001). This genus forages primarily under daylight conditions, with activity peaks in relatively dim but still photopic light shortly after sunrise and before sunset (Frederick and Bildstein 1992). *Eudocimus’* retinas have a higher proportion of cones than rods, compatible with a visual system adapted to bright rather than low-light environments (Rojas et al. 1997). As other ibises, *Eudocimus* relies on bill-probing to find concealed prey (Hancock et al. 2010). Vision contributes only marginally to its feeding behaviour, and is more important for predator detection and flight (Martin 2017). *Apteribis’* ancestors had likely a similar foraging ecology, relying predominantly on non-visual sensory inputs. Flightlessness can evolve rapidly on islands and is promoted by the lack of predators (Slikas et al. 2002; Torres and Clarke 2018). Such conditions correspond to the Hawaiian islands before human arrival (James et al. 1987; Olson and James 1982; 1984; Steadman 1995) and relaxation of the visual constraints associated with flight was likely a predisposing factor facilitating the reduced reliance on vision in *Apteribis*, as well as in another Hawaiian species (*Talpanas lippa*, Iwaniuk et al. 2009; Witmer et al. 2017). The combination of flightlessness, a tactile foraging strategy, and likely increased prey availability at night favoured a downscaling of the visual system of *Apteribis* rather than an investment in specializations for nocturnality, such as the enlarged eyes found in owls and caprimulgids (Hall and Ross 2007).

Given the reduced visual system in *Apteribis*, this genus likely relied on non-visual sensory modalities, such as tactile or olfactory cues, to orient itself and forage under low light conditions. All ibises and spoonbills rely on tactile foraging and possess a bill-tip organ that enables them to find prey buried in the substrate without the need for visual or olfactory input (Cunningham et al. 2010a; Cunningham et al. 2010b; du Toit et al. 2022). The somatosensory system of *Apteribis* is not significantly enlarged compared to that of other ibises (Fig. 4B, C, D), which suggests that its bill tip organ provided sufficiently high acuity and sensitivity to support foraging at night. That said, our examination of bill morphology suggests that *Apteribis* sp. may comprise two distinct taxa, or at least two “ecomorphs.” As shown in Fig. 4A, we identified a previously unrecognized morphological feature that was present in some, but not all, *Apteribis sp*. specimens: a double-row of pits on the lingual side of the mandibles. This feature is not known to occur in any other ibis species and suggests that *Apteribis* might be even more diverse than previously recognized. A double row of pits would likely increase the acuity and sensitivity of the bill tip organ, allowing those individuals to exploit other prey items or a different environment.

Although *Apteribis* is not an outlier in somatosensory anatomy, its number of bill tip organ pits and size of the trigeminal nerve are most similar to that of the sacred ibises (*Threskiornis* spp.) and Crested ibis (*Nipponia nippon*) than its putative relative (*Eudocimus*) (Figs. 4B, C). Interestingly, both *Nipponia* and *Threskiornis* have highly plastic foraging behaviour. *Nipponia* typically probes in mud or shallow water, but also picks up prey directly from the ground surface (Billerman et al. 2022; Hancock et al. 2010). *Threskiornis* species have the broadest foraging behaviour of any of the ibises; they will feed in marshes, flooded grasslands, and drier earth, as well as pecking at carrion and directly from the ground surface, including garbage in urban environments in Australia (Billerman et al. 2022; Clergeau and Yesou 2006; Ding 2004; Hancock et al. 2010; Lowe 1984). Given the similarity in the somatosensory anatomy among these genera, we suspect that *Apteribis* was able to exploit a variety of habitats. While *Apteribis* specimens have been primarily recovered from drier regions of the islands (Olson and James 1991; Olson and Wetmore 1976), this pattern may simply reflect sampling bias, as remains in these areas can be more easily detected or exposed. It is plausible that a plastic foraging strategy would have been advantageous for a flightless, island-confined genus (Diamond 1970; Keast 1970; Scott et al. 2003; Whittaker and Fernández-Palacios 2007). Key components in the diet of extant ibises are larval insect stages, land snails, aquatic crustaceans and small mollusks (Billerman et al. 2022; Hancock et al. 2010). In Hawaii, Amastridae snails are an endemic group known for their extreme species richness and ground-dwelling nocturnal habits (Cowie, Evenhuis et al. 1995; Régnier, Bouchet et al. 2015). While direct evidence is lacking, we hypothesize that these snails were likely a preferred food source for *Apteribis*, in agreement with (Olson and James 1991). Further, the nocturnal activity of these snails may have been among the key selective pressures that drove *Apteribis* into a nocturnal niche. Other possible prey include the larval stages of lepidopteran, dipteran and coleopteran insects. Of particular interest are *Hyposmocoma* moth larvae, which occupy nearly all habitat types across the Hawaiian archipelago, including within the beds of fast-flowing streams on Kauai, Oʻahu, Molokaʻi, and Maui (Rubinoff 2008).

Olfaction is another sensory modality that is often enhanced in some nocturnal species to aid in foraging, social, and other behaviour (Corfield et al. 2014; Corfield et al. 2015; Cunningham et al. 2009; Hagelin 2004; Le Duc et al. 2015; Martin et al. 2007). However, neither the width of the olfactory sulcus nor surface area of the olfactory bulbs differed in *Apteribis* compared with other ibises. Delineating the precise borders of the olfactory bulbs can be challenging in ibises, as well as many other bird clades, but even a qualitative assessment of the endocasts indicated that the olfactory bulbs of *Apteribis* do not appear to differ from other species (Figs. 2, 5B). What olfactory cues ibises can detect or what they use olfaction for has yet to be fully investigated. One experiment showed that Madagascar Ibis (*Lophotibis cristata*) does not need olfactory cues to detect prey (Cunningham et al. 2010b), but it remains unclear to what extent olfaction is used for foraging versus other functions, such as navigation, social behaviour, and kin or mate recognition in ibises (Hagelin and Jones 2007).

The picture that emerges from our analyses is that *Apteribis* was a nocturnal, flightless ibis with plastic foraging behaviour similar to *Threskiornis* and *Nipponia*. This would have allowed *Apteribis* to take advantage of abundant, nocturnal snails and aquatic larvae as a food source and be flexible in their habitat and foraging behaviour. To some extent, this niche is similar to that of kiwi and the mole-duck *Talpanas* (Cunningham et al. 2013; Iwaniuk et al. 2009; Martin et al. 2007; Witmer et al. 2017); nocturnal, tactile-foraging species that evolved on islands free of mammalian predators. The mole-duck is only known from Kauai (Iwaniuk et al. 2009), an island within the Hawaiian archipelago that lacked *Apteribis* (Olson and James 1991), suggesting that they might have been occupying similar niches on different islands. Many aspects of kiwi anatomy, behaviour, and ecology are divergent from almost all other birds, including having the most dramatic reduction in visual system of any bird (Garamszegi et al. 2002; Hall and Ross 2007; Martin et al. 2007) and an unusual expansion and anatomy of the olfactory bulbs (Corfield et al. 2014). Nevertheless, kiwi are nocturnal, beak-probing birds living on oceanic islands that were historically free of mammalian predators, so there are at least some similarities with *Apteribis*. If we also include *Talpanas* as another nocturnal, tactile specialist, this suggests that the evolution of a flightless, nocturnal niche that relies less on vision and more on a tactile foraging is not unique to New Zealand, but is a pattern repeated across several oceanic islands. This once more emphasizes the importance of studying species on oceanic islands as a means of understanding evolutionary patterns and principles.

## Author contributions

Sara Citron (data curation, formal analysis, investigation, visualization, writing – original draft), Aubrey Keirnan (writing – review and editing), Vera Weisbecker (resources, writing – review and editing), Helen James (investigation, supervision, writing – review and editing), Andrew N. Iwaniuk (conceptualization, funding acquisition, project administration, resources, software, supervision, writing – review and editing).

## Supplementary Material

icaf159_Supplemental_Files

## Data Availability

The data underlying this article are available in the article and in its online supplementary material.

## References

[bib118_280_310625] Andrianarimisa A, Razafimanjato G. 2008. Madagascar Sacred Ibis Threskiornis bernieri: current population status, distribution, and implications for conservation. PAOC. 120–130.

[bib1] Balanoff AM, Bever G, Colbert MW, Clarke JA, Field DJ, Gignac PM, Ksepka DT, Ridgely RC, Smith NA, Torres CR. 2016. Best practices for digitally constructing endocranial casts: examples from birds and their dinosaurian relatives. J Anat. 229:173–90. 26403623 10.1111/joa.12378 PMC4948053

[bib2] Baumel JJ . 1993. Handbook of avian Anatomy. Cambridge, Massachusetts: Nuttall Ornithological Club. p. 779.

[bib3] Berkhoudt H . 1979. The morphology and distribution of cutaneous mechanoreceptors (herbst and grandry corpuscles) in bill and tongue of the mallard (Anas platyrhynchos l.). Neth J Zool. 30:1–34.

[bib4] Billerman S, Keeney B, Rodewald P, Schulenberg T. 2022. Birds of the World. Ithaca, new york, USA: Cornell laboratory of ornithology.

[bib5] Blondel J . 2000. Evolution and ecology of birds on islands: trends and prospects. Vie Et Milieu/Life & Environment. 50:205–20.

[bib6] Boyer AG, Jetz W. 2010. Biogeography of body size in pacific island birds. Ecography. 33:369–79.

[bib116_662_315725] Boyer AG . 2008. Extinction patterns in the avifauna of the Hawaiian islands. Divers. Distrib. 14:509–517.

[bib7] Butler AB, Hodos W. 2005. Comparative Vertebrate Neuroanatomy: Evolution and Adaptation. New York, New York, USA: John Wiley & Sons. p. 752.

[bib8] Catania KC . 2005. Evolution of sensory specializations in insectivores. Anat Rec A. 287:1038–50. 10.1002/ar.a.20265 16215983

[bib9] Caves EM, Brandley NC, Johnsen S. 2018. Visual acuity and the evolution of signals. Trends Ecol. Evol. 33:358–72. 29609907 10.1016/j.tree.2018.03.001

[bib10] Caves EM, Fernández-Juricic E, Kelley LA. 2024. Ecological and morphological correlates of visual acuity in birds. J. Exp. Biol. 227:jeb246063. 38126722 10.1242/jeb.246063 PMC10906485

[bib11] Clegg S . 2010. Evolutionary changes following island colonization in birds. In: Losos J. B., Ricklefs R. E. Princeton, New Jersey, USA: Princeton University Press. The theory of island biogeography revisited. p. 293–325.

[bib12] Clergeau P, Yesou P. 2006. Behavioural flexibility and numerous potential sources of introduction for the sacred ibis: causes of concern in western europe?. Biol Invasions. 8:1381–8.

[bib13] Cobb S . 1968. The size of the olfactory bulb in 108 species of birds. Auk. 85:55–61.

[bib14] Corfield JR, Eisthen HL, Iwaniuk AN, Parsons S. 2014. Anatomical specializations for enhanced olfactory sensitivity in kiwi, apteryx mantelli. Brain Behav. Evol. 84:214–26. 25376305 10.1159/000365564

[bib15] Corfield JR, Gsell AC, Brunton D, Heesy CP, Hall MI, Acosta ML, Iwaniuk AN. 2011. Anatomical specializations for nocturnality in a critically endangered parrot, the kakapo (Strigops habroptilus). PLoS One. 6:e22945. 21860663 10.1371/journal.pone.0022945 PMC3157909

[bib16] Corfield JR, Price K, Iwaniuk AN, Gutiérrez-Ibáñez C, Birkhead T, Wylie DR. 2015. Diversity in olfactory bulb size in birds reflects allometry, ecology, and phylogeny. Front. Neuroanat. 9:102. 26283931 10.3389/fnana.2015.00102 PMC4518324

[bib120_605_311425] Cowie RH, Evenhuis NL, Christensen CC. 1995. Catalog of the native land and freshwater molluscs of the Hawaiian Islands.

[bib17] Crole MR, Soley JT. 2016. Comparative morphology, morphometry and distribution pattern of the trigeminal nerve branches supplying the bill tip in the ostrich (s truthio camelus) and emu (d romaius novaehollandiae). Acta Zool. 97:49–59.

[bib18] Cunningham S, Castro I, Alley M. 2007. A new prey-detection mechanism for kiwi (Apteryx spp.) suggests convergent evolution between paleognathous and neognathous birds. J Anat. 211:493–502. 17711422 10.1111/j.1469-7580.2007.00786.x PMC2375824

[bib19] Cunningham SJ, Alley MR, Castro I, Potter MA, Cunningham M, Pyne MJ. 2010a. Bill morphology of ibises suggests a remote-tactile sensory system for prey detection. Auk. 127:308–16.

[bib20] Cunningham SJ, Castro I, Jensen T, Potter MA. 2010b. Remote touch prey-detection by madagascar crested ibises Lophotibis cristata urschi. J. Avian Biol. 41:350–3.

[bib21] Cunningham SJ, Castro I, Potter MA. 2009. The relative importance of olfaction and remote touch in prey detection by north island brown kiwis. Anim Behav. 78:899–905.

[bib22] Cunningham SJ, Corfield JR, Iwaniuk AN, Castro I, Alley MR, Birkhead TR, Parsons S. 2013. The anatomy of the bill tip of kiwi and associated somatosensory regions of the brain: comparisons with shorebirds. PLoS One. 8:e80036. 24244601 10.1371/journal.pone.0080036 PMC3828210

[bib23] Deacon TW . 1990. Fallacies of progression in theories of brain-size evolution. Int J Primatol. 11:193–236.

[bib24] Diamond JM . 1970. Ecological consequences of island colonization by southwest pacific birds, types of niche shifts. Proc Natl Acad Sci. 67:529–36. 16591871 10.1073/pnas.67.2.529 PMC283240

[bib25] Diamond JM . 1981. Flightlessness and fear of flying in island species. Nature. 293:507–8.

[bib26] Ding C . 2004. Research on the Crested ibis. Shanghai: Shanghai Scientific and Technological Educational Publishing House.

[bib27] Doutrelant C, Paquet M, Renoult JP, Grégoire A, Crochet PA, Covas R. 2016. Worldwide patterns of bird colouration on islands. Ecol Lett. 19:537–45. 26932367 10.1111/ele.12588

[bib28] Dove CJ, Olson SL. 2011. Fossil feathers from the hawaiian flightless ibis (Apteribis sp.): plumage coloration and systematics of a prehistorically extinct bird. J. Paleontol. 85:892–7.

[bib31] du Toit C, Bond AL, Cunningham SJ, Field D, Portugal SJ. 2024. Tactile bill-tip organs in seabirds suggests conservation of a deep avian symplesiomorphy. Biol. Lett. 20:20240259. 39288817 10.1098/rsbl.2024.0259 PMC11407862

[bib32] du Toit CJ, Chinsamy A, Cunningham SJ. 2022. Comparative morphology and soft tissue histology of the remote-touch bill-tip organ in three ibis species of differing foraging ecology. J. Anat. 241:966–80. 35938671 10.1111/joa.13734 PMC9482703

[bib29] Dubbeldam J, Veenman C. 1977. Studies on the somatotopy of the trigeminal system in the mallard, anas platyrhynchos l.: I. The ganglion trigeminale. Neth J Zool. 28:150–60.

[bib30] Dunning JB Jr . 2007. Crc Handbook of avian Body Masses. Boca Raton, Florida, USA: CRC press. p. 672.

[bib33] Early CM, Iwaniuk AN, Ridgely RC, Witmer LM. 2020a. Endocast structures are reliable proxies for the sizes of corresponding regions of the brain in extant birds. J. Anat. 237:1162–76. 32892372 10.1111/joa.13285 PMC7704230

[bib34] Early CM, Ridgely RC, Witmer LM. 2020b. Beyond endocasts: using predicted brain-structure volumes of extinct birds to assess neuroanatomical and behavioral inferences. Diversity. 12:34.

[bib35] Emerson B . 2002. Evolution on oceanic islands: molecular phylogenetic approaches to understanding pattern and process. Mol Ecol. 11:951–66. 12030975 10.1046/j.1365-294x.2002.01507.x

[bib36] Fasola M, Canova L. 1993. Diel activity of resident and immigrant waterbirds at Lake Turkana, Kenya. Ibis. 135:442–50.

[bib37] Fleischer RC, McIntosh CE. 2001. Molecular systematics and biogeography of the hawaiian avifauna. Stud. Avian Biol. 22:9.

[bib119_886_311025] Frederick PC, Bildstein KL. 1992. Foraging ecology of seven species of neotropical ibises(Threskiornithidae) during the dry season in the Llanos of Venezuela. Wilson bull. 104:1–21.

[bib38] Fröhlich A, Ducatez S, Neˇmec P, Sol D. 2024. Light conditions and the evolution of the visual system in birds. Evolution. 78:1237–47. 38558240 10.1093/evolut/qpae054

[bib39] Fromm A, Meiri S. 2021. Big, flightless, insular and dead: characterising the extinct birds of the quaternary. J Biogeogr. 48:2350–9.

[bib41] Garamszegi LZ, Møller AP, Erritzøe J. 2002. Coevolving avian eye size and brain size in relation to prey capture and nocturnality. Proc. R. Soc. B. 269:961–7. 10.1098/rspb.2002.1967 PMC1690973 12028780

[bib40] Garamszegi LZ . 2014. Modern Phylogenetic Comparative Methods and Their Application in Evolutionary Biology: Concepts and Practice. Seville, Spain: Springer. p. 552.

[bib43] Gentle MJ, Breward J. 1986. The bill tip organ of the chicken (Gallus gallus var. Domesticus). J Anat. 145:79. 3429310 PMC1166494

[bib42] Gentle MJ . 1989. Cutaneous sensory afferents recorded from the nervus intramandibularis of gallus gallus var domesticus. J. Comp. Physiol. A. 164:763–74. 2724186 10.1007/BF00616748

[bib44] Gottschaldt K-M, Lausmann S. 1974. The peripheral morphological basis of tactile sensibility in the beak of geese. Cell Tissue Res. 153:477–96. 4442093 10.1007/BF00231542

[bib45] Grant P . 1965. The adaptive significance of some size trends in island birds. Evolution. 19:355–67.

[bib46] Grant P . 1965b. Plumage and the evolution of birds on islands. Syst. Zool. 14:47–52.

[bib47] Grant PR . 2001. Reconstructing the evolution of birds on islands: 100 years of research. Oikos. 92:385–403.

[bib48] Gutiérrez-Ibáñez C, Iwaniuk AN, Wylie DR. 2010. The independent evolution of the enlargement of the principal sensory nucleus of the trigeminal nerve in three different groups of birds. Brain Behav. Evol. 74:280–94. 10.1159/000270904 20051684

[bib50] Hagelin JC, Jones IL. 2007. Bird odors and other chemical substances: a defense mechanism or overlooked mode of intraspecific communication?. Auk. 124:741–61.

[bib49] Hagelin JC . 2004. Observations on the olfactory ability of the kakapo Strigops habroptilus, the critically endangered parrot of New Zealand. Ibis. 146:161–4.

[bib51] Hall M, Ross C. 2007. Eye shape and activity pattern in birds. J. Zool. 271:437–44.

[bib53] Hall MI, Iwaniuk AN, Gutiérrez-Ibáñez C. 2009. Optic foramen morphology and activity pattern in birds. Anatomical Rec. 292:1827–45. 10.1002/ar.21007 19777569

[bib52] Hall MI . 2008. The anatomical relationships between the avian eye, orbit and sclerotic ring: implications for inferring activity patterns in extinct birds. J. Anat. 212:781–94. 18510506 10.1111/j.1469-7580.2008.00897.x PMC2423400

[bib54] Hancock J, Kushlan JA, Kahl MP. 2010. Storks, Ibises and Spoonbills of the World. San Diego, CA: Academic Press. p. 382.

[bib55] Hume JP . 2017. Extinct Birds. London, UK: Bloomsbury Publishing. p. 544.

[bib56] Iwaniuk AN, Keirnan AR, Janetzki H, Mardon K, Murphy S, Leseberg NP, Weisbecker V. 2020. The endocast of the night parrot (pezoporus occidentalis) reveals insights into its sensory ecology and the evolution of nocturnality in birds. Sci. Rep. 10:9258. 32518353 10.1038/s41598-020-65156-0 PMC7283296

[bib57] Iwaniuk AN, Nelson JE, James HF, Olson SL. 2004. A comparative test of the correlated evolution of flightlessness and relative brain size in birds. J. Zool. 263:317–27.

[bib58] Iwaniuk AN, Olson SL, James HF. 2009. Extraordinary cranial specialization in a new genus of extinct duck (aves: anseriformes) from Kauai, Hawaiian islands. Zootaxa. 2296:47–67.

[bib59] Iwaniuk AN, Wylie DR. 2020. Sensory systems in birds: what we have learned from studying sensory specialists. J. Comp. Neurol. 528:2902–18. 32133638 10.1002/cne.24896

[bib60] James HF, Stafford TW Jr, Steadman DW, Olson SL, Martin PS, Jull A, McCoy PC. 1987. Radiocarbon dates on bones of extinct birds from hawaii. Proc Natl Acad Sci. 84:2350–4. 3470800 10.1073/pnas.84.8.2350 PMC304648

[bib61] Jetz W, Thomas GH, Joy JB, Redding DW, Hartmann K, Mooers AO. 2014. Global distribution and conservation of evolutionary distinctness in birds. Curr. Biol. 24:919–30. 24726155 10.1016/j.cub.2014.03.011

[bib62] Jezierski MT, Smith WJ, Clegg SM. 2024. The island syndrome in birds. J Biogeogr. 51:1607–22.

[bib63] Johnston P, Mitchell KJ. 2021. Contrasting patterns of sensory adaptation in living and extinct flightless birds. Diversity. 13:538.

[bib64] Keast A . 1970. Adaptive evolution and shifts in niche occupation in island birds. Biotropica. 2:61–75.

[bib65] Keirnan AR, Cunha F, Citron S, Prideaux G, Iwaniuk AN, Weisbecker V. 2025. Avian telencephalon and cerebellum volumes can be accurately estimated from digital brain endocasts. Biol. Lett. 21:20240596. 39837487 10.1098/rsbl.2024.0596 PMC11750377

[bib66] Kirch PV . 1911. When did the polynesians settle Hawai’i? A review of 150 years of scholarly inquiry and a tentative answer.

[bib67] Le Duc D, Renaud G, Krishnan A, Almén MS, Huynen L, Prohaska SJ, Ongyerth M, Bitarello BD, Schiöth HB, Hofreiter M. 2015. Kiwi genome provides insights into evolution of a nocturnal lifestyle. Genome Biol. 16:147. 26201466 10.1186/s13059-015-0711-4 PMC4511969

[bib68] Lewis D . 2018. Belonging on an Island: Birds, Extinction, and Evolution in hawaiʻi. New Haven, Connecticut: Yale University Pressp. 306.

[bib69] Li W, Liu D, Liao Y, He K, Wang C. 2025. Seasonal variation in nocturnal roost timing and diurnal movement in endangered crested ibis (Nipponia nippon): an adaptation strategy to environmental changes. Biology. 14:1496. 41300288 10.3390/biology14111496 PMC12650463

[bib70] Longrich NR, Olson SL. 2011. The bizarre wing of the jamaican flightless ibis xenicibis xympithecus: a unique vertebrate adaptation. Proc. R. Soc. B. 278:2333–7. 10.1098/rspb.2010.2117 PMC3119002 21208965

[bib71] Losos JB, Ricklefs RE. 2009. Adaptation and diversification on islands. Nature. 457:830–6. 19212401 10.1038/nature07893

[bib72] Lowe KW . 1984. The Feeding and Breeding Biology of the Sacred ibis Threskiornis aethiopicus in Southern Victoria. PhD thesis. Australia: University of Melbourne.

[bib75] Martin GR, Wilson K-J, Wild JM, Parsons S, Kubke MF, Corfield J. 2007. Kiwi forego vision in the guidance of their nocturnal activities. PLoS One. 2:e198. 17332846 10.1371/journal.pone.0000198 PMC1805817

[bib73] Martin GR . 1993. Producing the Image. Vision, Brain, and Behavior in Birds. Princeton, New Jersey: Princeton University Press. p. 5–24.

[bib74] Martin GR . 2017. What drives bird vision? Bill control and predator detection overshadow flight. Front. Neurosci. 11:619. 29163020 10.3389/fnins.2017.00619 PMC5682009

[bib76] McGinness HM, Lloyd-Jones LR, Robinson F, Hawken M, Cook D, O’Neill LG, Rapley S, Jackson MV, Piper M, Davies M et al. 2025. Satellite telemetry informs nesting ecology and management of nomadic ibis and spoonbills (Threskiornithidae) in remote breeding sites. Ornithol. Appl.:duaf053.

[bib77] McNab BK . 1994. Energy conservation and the evolution of flightlessness in birds. The Am. Nat. 144:628–42.

[bib79] Mpodozis J, Letelier JC, Concha ML, Maturana H. 1995. Conduction velocity groups in the retino-tectal and retino-thalamic visual pathways of the pigeon (Columba livia). Int. J. Neurosci. 81:123–36. 7775067 10.3109/00207459509015304

[bib80] Nebel , Elner. 2005. Functional association of bill morphology and foraging behaviour in calidrid sandpipers. Anim. Biol. 55:235–43.

[bib82] Olson SL, James HF. 1982. Fossil birds from the hawaiian islands: evidence for wholesale extinction by man before western contact. Science. 217:633–5. 17817532 10.1126/science.217.4560.633

[bib83] Olson SL, James HF. 1984. The role of polynesians in the extinction of the avifauna of the hawaiian islands. Quat. Extinct. 768–80.

[bib84] Olson SL, James HF. 1991. Descriptions of thirty-two new species of birds from the hawaiian islands: part I. Non-passeriformes. Ornithol. Monogr.:1–88.

[bib86] Olson SL, Wetmore A. 1976. Preliminary diagnoses of two extraordinary new genera of birds from pleistocene deposits in the hawaiian islands. Proc. Biol. Soc. Wash. 89:247–258.

[bib81] Olson SL . 1989. Extinction on islands: man as a catastrophe. Conservation Biology for the Next Century. Oxford, UK: Oxford Academic press. p. 50.

[bib87] Olson VA, Davies RG, Orme CDL, Thomas GH, Meiri S, Blackburn TM, Gaston KJ, Owens IP, Bennett PM. 2009. Global biogeography and ecology of body size in birds. Ecol Lett. 12:249–59. 19245587 10.1111/j.1461-0248.2009.01281.x

[bib88] Pagel M . 1999. Inferring the historical patterns of biological evolution. Nature. 401:877–84. 10553904 10.1038/44766

[bib89] Paradis E, Schliep K. 2019. Ape 5.0: an environment for modern phylogenetics and evolutionary analyses in r. Bioinformatics. 35:526–8. 30016406 10.1093/bioinformatics/bty633

[bib90] Paterno GB, Penone C, Werner GD. 2018. Sensiphy: an r-package for sensitivity analysis in phylogenetic comparative methods. Methods Ecol. Evol. 9:1461–7.

[bib91] Pearse WD, Davies JD, Wolkovich E. 2023. How to define, use, and interpret pagel’s lambda (lambda) in ecology and evolution. Biorxiv. 34:e70012.

[bib92] Pettigrew J, Frost B. 1985. A tactile fovea in the scolopacidae?. Brain Behav. Evol. 26:185–95. 4084760

[bib93] Pinheiro J, Bates D, DebRoy S, Sarkar D. 2012. Nonlinear mixed-effects models. R Package Version. 3:1–89.

[bib117_204_310125] Potier S, Mitkus M, Kelber A. 2020. Visual adaptations of diurnal and nocturnal raptors. Semin Cell Dev Biol. 106:116–126. 32654971 10.1016/j.semcdb.2020.05.004

[bib94] Profico A, Buzi C, Melchionna M, Veneziano A, Raia P. 2020. Endomaker, a new algorithm for fully automatic extraction of cranial endocasts and the calculation of their volumes. Am. J. Phys. Anthropol. 172:511–5. 32187657 10.1002/ajpa.24043

[bib95] Ramirez J, Miyaki CY, Del Lama S. 2013. Molecular phylogeny of Threskiornithidae (Aves: pelecaniformes) based on nuclear and mitochondrial DNA. Gen Mol Res. 12:2740–50. 10.4238/2013.July.30.11 23979898

[bib121_338_311825] Régnier C, Bouchet P, Hayes KA, Yeung NW, Christensen CC, Chung DJ, Cowie RH. 2015. Extinction in a hyperdiverse endemic Hawaiian land snail family and implications for the underestimation of invertebrate extinction. Conserv. Biol. 29:1715–1723. 26234768 10.1111/cobi.12565

[bib96] Riamon S, Balouet J-C, Rolland-Guillard J, Salaviale C, Guenser P, Steyer J-S, Louchart A. 2022. The endocast of the insular and extinct Sylviornis neocaledoniae (Sves, Salliformes), reveals insights into its sensory specializations and its twilight ecology. Sci. Rep. 12:21185. 36477415 10.1038/s41598-022-14829-z PMC9729198

[bib97] Rojas L-M, McNeil R, Cabana T, Lachapelle P. 1997. Diurnal and nocturnal visual function in two tactile foraging waterbirds: the American white Ibis and the black skimmer. Condor. 99:191–200.

[bib98] Rubinoff D . 2008. Phylogeography and ecology of an endemic radiation of hawaiian aquatic case-bearing moths (Hyposmocoma: cosmopterigidae). Philos. Trans. R. Soc. B. 363:3459–65. 10.1098/rstb.2008.0115 PMC2607376 18765359

[bib99] Sayol F, Downing PA, Iwaniuk AN, Maspons J, Sol D. 2018. Predictable evolution towards larger brains in birds colonizing oceanic islands. Nat. Commun. 9:2820. 30065283 10.1038/s41467-018-05280-8 PMC6068123

[bib100] Sayol F, Steinbauer MJ, Blackburn TM, Antonelli A, Faurby S. 2020. Anthropogenic extinctions conceal widespread evolution of flightlessness in birds. Sci. Adv. 6:eabb6095. 33268368 10.1126/sciadv.abb6095 PMC7710364

[bib101] Scott SN, Clegg SM, Blomberg SP, Kikkawa J, Owens IP. 2003. Morphological shifts in island-dwelling birds: the roles of generalist foraging and niche expansion. Evolution. 57:2147–56. 14575334 10.1111/j.0014-3820.2003.tb00392.x

[bib102] Slikas B, Olson SL, Fleischer RC. 2002. Rapid, independent evolution of flightlessness in four species of Pacific Island rails (Rallidae): an analysis based on mitochondrial sequence data. J. Avian Biol. 33:5–14.

[bib103] Smaers J, Mongle C. 2014. Evomap: r package for the evolutionary mapping of continuous traits. GitHub. (https://github com/JeroenSmaers/evomap).

[bib104] Steadman DW . 1995. Prehistoric extinctions of pacific island birds: biodiversity meets zooarchaeology. Science. 267:1123–31. 17789194 10.1126/science.267.5201.1123

[bib105] Steadman DW . 2006. Extinction and Biogeography of Tropical Pacific Birds. Chicago, Illinois: University of Chicago Press. p. 480.

[bib106] Team RDC . 2010. R: a Language and Environment for Statistical Computing. R Found. Stat. Comput. 1:409.

[bib107] Tobias JA, Sheard C, Pigot AL, Devenish AJ, Yang J, Sayol F, Neate-Clegg MH, Alioravainen N, Weeks TL, Barber RA. 2022. Avonet: morphological, ecological and geographical data for all birds. Ecol. Lett. 25:581–97. 35199922 10.1111/ele.13898

[bib108] Torres CR, Clarke JA. 2018. Nocturnal giants: evolution of the sensory ecology in elephant birds and other palaeognaths inferred from digital brain reconstructions. Proc. R. Soc. B. 285:20181540. 10.1098/rspb.2018.1540 PMC6235046 30381378

[bib109] Wackermannová M, Pinc L, Jebavý L. 2016. Olfactory sensitivity in mammalian species. Physiol. Res. 65:369. 27070753 10.33549/physiolres.932955

[bib110] Warren BH, Simberloff D, Ricklefs RE, Aguilée R, Condamine FL, Gravel D, Morlon H, Mouquet N, Rosindell J, Casquet J. 2015. Islands as model systems in ecology and evolution: prospects fifty years after macarthur-wilson. Ecol. Lett. 18:200–17. 25560682 10.1111/ele.12398

[bib111] West AK, Xu EM, Nelson MD, Hart TR, Stricker EM, Cones AG, Martin GM, Strickland K, Lambert DL, Burman L. 2022. Quantitative evaluation of tactile foraging behavior in pekin and muscovy ducks. Front. Physiol.. 13:921657. 35774281 10.3389/fphys.2022.921657 PMC9237358

[bib112] West B . 2014. New observations of the andean ibis (Theristicus branickii, Threskiornithidae): distribution, movements, and behavior near volcan antisana. Independent Study Project Collection.

[bib113] Whittaker RJ, Fernández-Palacios JM. 2007. Island Biogeography: Ecology, Evolution, and Conservation. Oxford, United Kingdom: Oxford University Press. p. 401.

[bib114] Witmer LM, Ridgely RC, James HF, Olson SL, Iwaniuk AN. 2017. The remarkable, recently extinct “mole-duck” Talpanas lippa (aves: anseriformes) from Kauai, Hawaii: behavioral implications of its neuroanatomy and skull morphology. FASEB J. 31:251–6.

[bib115] Wright NA, Steadman DW, Witt CC. 2016. Predictable evolution toward flightlessness in volant island birds. Proc Natl Acad Sci USA. 113:4765–70. 27071105 10.1073/pnas.1522931113 PMC4855539

[bib116] Wylie DR, Gutierrez-Ibanez C, Pakan JM, Iwaniuk AN. 2009. The optic tectum of birds: mapping our way to understanding visual processing. Can. J. Exp. Psychol. 63:328. 20025392 10.1037/a0016826

[bib117] Ziolkowski LH, Gracheva EO, Bagriantsev SN. 2022. Tactile sensation in birds: physiological insights from avian mechanoreceptors. Curr. Opin. Neurobiol. 74:102548. 35489134 10.1016/j.conb.2022.102548 PMC9167745

