# Correction to: Comparative Anatomy Supports the Evolution of Nocturnality in the Extinct Hawaiian Ibis Apteribis

**DOI:** 10.1093/icb/icag091

**Published:** 2026-06-24

This is a correction to: Sara Citron, Aubrey Keirnan, Vera Weisbecker, Helen James, Andrew N Iwaniuk, Comparative Anatomy Supports the Evolution of Nocturnality in the Extinct Hawaiian Ibis *Apteribis, Integrative and Comparative Biology*, Volume 66, 2026, icaf159, https://doi.org/10.1093/icb/icaf159

In the originally published version of this manuscript, there were erroneous specimen numbers contained within the supplementary materials.

Four entries for ‘Ajaia’ have been corrected to read “USNM635736” instead of “USNM365736”.

Two entries for ‘Bostrichia’ have been corrected to read “USNM634733” instead of “USNM643733”.

