# Supplementary figures and images for: Comparative Anatomy Supports the Evolution of Nocturnality in the Extinct Hawaiian Ibis Apteribis
Source: Integr Comp Biol. 2025 Dec 19;66:icaf159. doi: 10.1093/icb/icaf159 (PMC12805921; doi:10.1093/icb/icaf159)

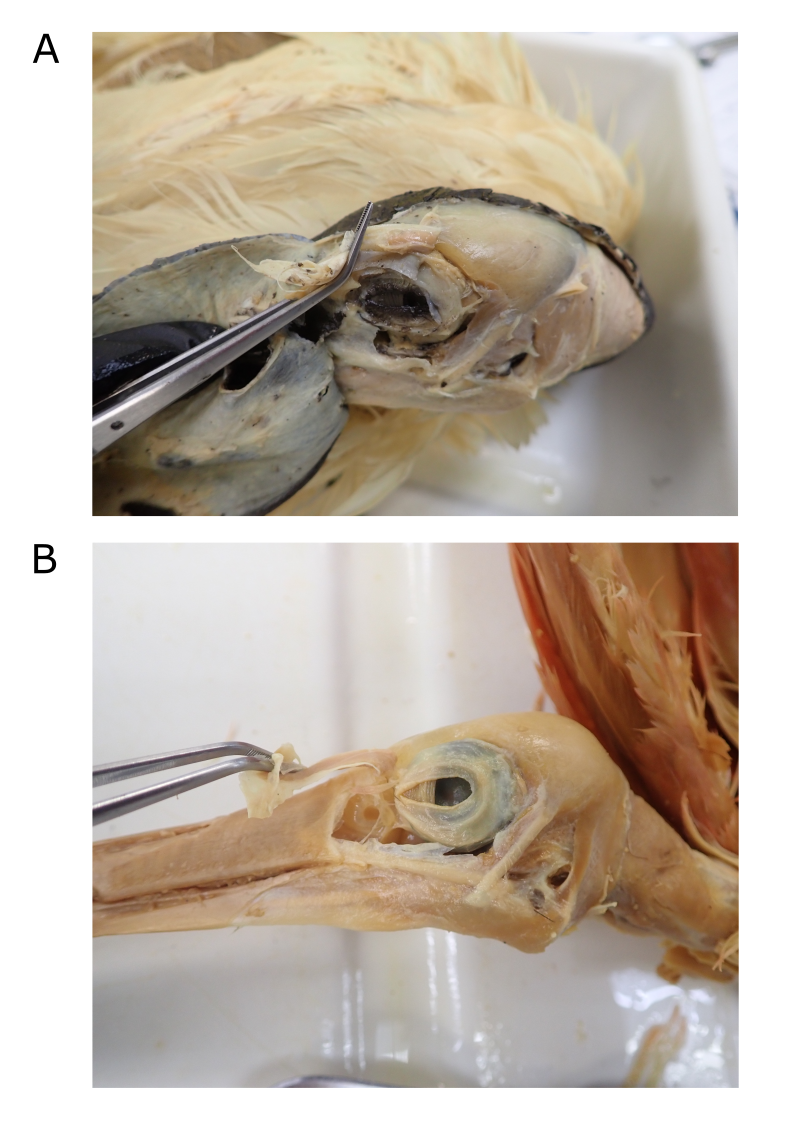

Supplement: icaf159_Supplemental_Files [file icaf159_supplemental_files.zip › icb-2025-0172-File008.png]

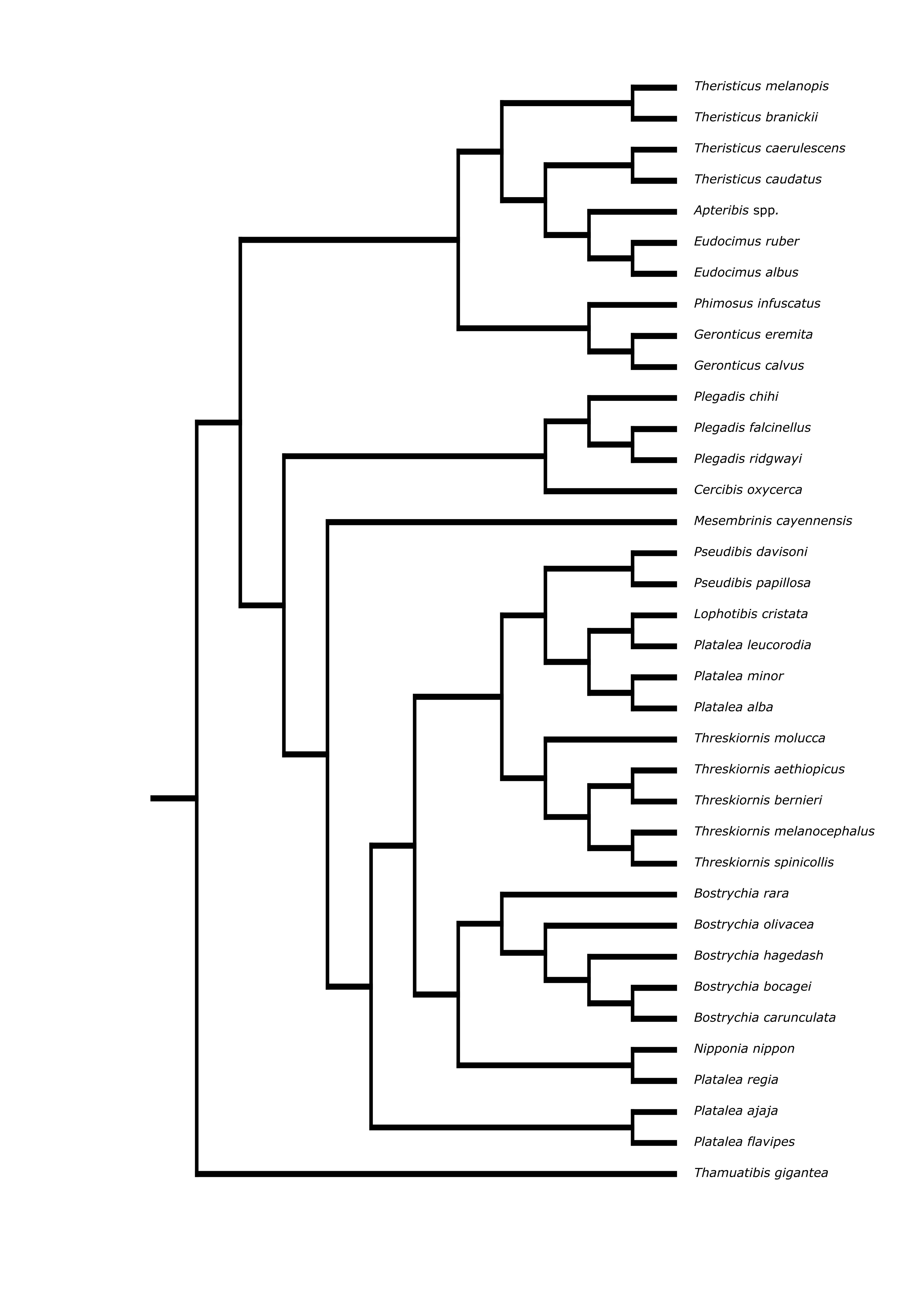

Supplement: icaf159_Supplemental_Files [file icaf159_supplemental_files.zip › icb-2025-0172-File009.png]
